# Inheritance of Diapause in Crosses between the Northernmost and the Southernmost Strains of the Asian Corn Borer *Ostrinia furnacalis*

**DOI:** 10.1371/journal.pone.0118186

**Published:** 2015-02-23

**Authors:** Shu Fu, Chao Chen, Liang Xiao, Haimin He, Fangsen Xue

**Affiliations:** Institute of Entomology, Jiangxi Agricultural University, Nanchang, Jiangxi Province, China; United States Department of Agriculture (USDA), UNITED STATES

## Abstract

The northernmost Harbin strain (N strain) of the Asian corn borer, *Ostrinia furnacalis* enters facultative diapause as fully grown larvae in response to short daylengths; whereas the southernmost Ledong strain (S strain) exhibits almost no diapause under the same light conditions. In the present study, we examined the inheritance of diapause induction and termination by crossing the two strains under a range of environmental conditions. The N strain showed a typical long-day response with a critical daylength of approximately15.88 h at 22°C, 15.72 h at 25°C and 15.14 h at 28°C, whereas the S strain showed a weak photoperiodic response at 22°C. The F1 progeny also showed a long-day response at 22, 25 and 28°C. However, the critical daylengths in S ♀ × N ♂ crosses were significantly longer than those in N ♀ × S ♂ crosses, indicating a sex linkage in the inheritance of diapause induction, with the male parent having more influence on the following F1 progeny. The incidence of diapause in S ♀ × N ♂ crosses was the same as in the N strain under short daylengths of 11-13 h, indicating that diapause trait is completely dominant over the non-diapause trait. The critical daylength in backcross to N was significantly longer than it was in backcross to S, showing a grandfather gene effect. Whether the inheritance of diapause fits an additive hypothesis or not was dependent on the rearing photoperiod, and the capacity for diapause was transmitted genetically in the manner of incomplete dominance. The duration of diapause for the reciprocal crosses under different diapause-terminating conditions showed different patterns of inheritance. The results in this study reveal that genetic and genetic-environmental interactions are involved in diapause induction and termination in *O. furnacalis*.

## Introduction

Diapause is an endocrine controlled mechanism by which an organism avoids the effects of a temporarily hostile environment during certain stages of its life history [1]. The occurrence of diapause is determined by various environmental inputs, but most insects in temperate regions use photoperiod as the primary cue for monitoring seasonal changes [2, 3]. Geographical strains that differ in their diapause characteristics are common among insect species [3–5]. A latitudinal cline in the incidence of diapause, in which the strains of insect species from temperate regions undergo photoperiodic induction of diapause but the strains inhabiting tropical regions are entirely free of diapause, or only a few individuals enter diapause under conditions of short daylength and low temperature, has been reported in a number of insects, such as the pink bollworm, *Pectinophora gossypiella* [6,7], the flesh fly, *Sarcophaga peregrina* [8], the two-spotted spider mite, *Tetranychus urticae* [9,10] and the cotton bollworm, *Helicoverpa armigera* [11]. Such populations usually inhabit rather broad and distinct geographical regions. Understanding how seasonal adaptations are inherited has important and direct implications for both theoretical and applied biology. As a component of pest management, the seasonal asynchrony caused by crossing a non-diapause tropical strain with a temperate strain could be introduced into pest populations [12,13]. That is, an introduction of low-diapause genes into a temperate population would radically reduce the incidence of diapause, thus seriously affecting the overwintering capabilities of the population.

The genetic aspects of diapause in the previously mentioned species have been reported to some extent. In *P*. *gossypiella*, crosses between the non-diapause strain from southern India and two diapause strains from Arizona (U.S.A.) indicated that the F_1_ progeny expressed a diapause response that was intermediate of the two parents and the progeny from the male parent of the India strain usually had a lower incidence of diapause. Such sex related differences were more evident in F_2_ progeny and backcrosses [7]. Reciprocal crosses made between a non-diapause strain of the flesh fly, *S*. *peregrine* from New Guinea and a diapause strain from Japan under the short daylengths of 9, 10, 11 h at 20°C showed that diapause was suppressed in 30–40% of F_1_ progeny, suggesting polygenic inheritance of diapause induction [8]. Crosses between the southernmost non-diapause strain of the spider mite, *T*. *pueraricola*, and two diapause strains showed that non-diapause was a dominant characteristic over diapause and that the characteristic was controlled by simple Mendelian inheritance [14]. Crosses of the diapause strain of the two-spotted spider mite, *T*. *urticae*, from northern Japan with two non-diapause strains from southern Japan, and the reciprocal crosses and backcrosses to the non-diapause strains revealed that the non-diapause phenotype was inherited in a completely dominant manner, suggesting that dominant non-diapause alleles control the non-diapause phenotype [10]. Crosses between the southernmost non-diapause strain from the tropical region and the temperate diapause strain in *H*. *armigera* revealed that there was a sexual asymmetry in the incidence of diapause in reciprocal crosses, with the male parent exerting a greater effect on diapause incidence than the female parent, and that the capacity for diapause was transmitted genetically in the manner of incomplete dominance, with the non-diapause characteristic partially dominant over the diapause characeteristic [11]. It is widely accepted that such differences in diapause potential are due to the considerable genetic polymorphism that is formed by selective forces [4].

The Asian corn borer, *Ostrinia furnacalis* (Guenée) (Lepidoptera: Crambidae), is widely distributed in corn producing regions throughout China. The larvae can cause significant damage to their main host, corn (*Zea mays* L.) [15]. Due to the geographic variation in the life history and diapause response of *O*. *furnacalis*, the northernmost Harbin strain (N strain) mainly produces one generation per year [16] and enters a photoperiodically induced larval diapause as fully grown larvae during autumn in response to high temperatures during the summer [17]; whereas the southernmost Ledong strain (S strain), produces seven generations per year in Ledong [18] and only a few larvae enter diapause in response to short daylengths and low temperatures in laboratory conditions [19]. In the present study, the two strains were crossed to study the inheritance of diapause induction and termination. The objective was to inspect the genetic components of diapause induction under a broad range of photoperiod and temperature ranges by determining the incidence of diapause in the two distinct Asian corn borer ecotypes, their reciprocal hybrids, F_2_ crosses, and backcross progeny. The effect of the genetic background of each parent on larval diapause duration was also tested.

## Materials and Methods

### Ethics Statement

Because the Asian corn borer, *O*. *furnacalis* is one of the most economically important pests in corn producing regions throughout China, no permits were required for collecting the insect and performing the experiments. All experiments were carried out at the Institute of Entomology, Jiangxi Agricultural University, Nanchang, Jiangxi province (28°46′ N, 115°49′ E).

### Strains of *O*. *furnacalis*

The N and S strains were collected from corn-fields as adults in early July 2013 in the two geographical regions of Harbin city, Heilongjiang Province (44.9° N, 127.2° E) and Ledong county, Hainan province (18.5° N, 108.9° E), respectively. Adults were placed into plastic bags with 10% honey-water to produce egg masses. After hatching, larvae were transferred to plastic boxes (diameter 12 cm, height 15 cm) and reared on an artificial diet [20] under a diapause-averting photoperiod of LD 18:6 h at 25°C until pupation. Pupae were placed individually in 24-well cell culture plates (for each well: diameter 1.5 cm, height 2 cm) for eclosion and maintained under LD 18:6 h at 25°C. Adults were sexed on the day of eclosion, and females were allowed to mate with males from either the same strain or a different strain.

### Crosses

Pure strains and reciprocal parental crosses were made as follows: N × N (females shown on the left), S × S, N × S and S × N. At least 80 pairs were used in each cross. The progeny of these crosses were reared under different photoperiods at 22, 25 and 28°C. Unless otherwise stated, each experimental unit was tested by rearing three replicates of at least 50 newly hatched larvae and observing the incidence of pupation and diapause when the insects reached maturity. The incidence of diapause was determined based on the proportion of mature larvae that failed to pupate within two weeks after comparable control cultures had completed pupation [19].

Backcrosses and hybrids for F_1_ crosses were obtained from non-diapause parents that were reared under LD 18:6 h at 25°C. When these adults had just emerged, the virgin females and males were paired as follows: (N × S) × (N × S), (S × N) × (S × N), N × (N × S), N × (S × N), (N × S) × N, (S × N) × N, S × (N × S), S × (S × N), (S × N) × S, (N × S) × S, N × N, S × S, (S × N) × (N × S) and (N × S) × (S × N). The progeny of these crosses were reared under different photoperiods at 25°C. Each experimental unit was tested by rearing three replicates of at least 50 newly hatched larvae

All of the equations used for the present analyses were as described by Henrich and Denlinger [21]. In brief, the expected incidences of diapause (*E*) for an additive model of a backcross (Bc) to the N strain (Bc-N), S strain (Bc-S), and F_2_ were generated with the equations:

$$E_{\text{Bc}-\text{N}}=(O_{\text{N}}+O_{\text{F}1})/2$$

$$E_{\text{Bc}-\text{S}}=(O_{\text{S}}+O_{\text{F}1})/2$$

$$E_{\text{F}2}=(O_{\text{N}}+2O_{\text{F}1}+O_{\text{S}})/4$$

where *O* indicates the observed incidence of diapause of the designated population. If the observed and expected values in the F_2_ group are equal:

$$E_{\text{F}2}=O_{\text{F}2}$$

$$then\;\;4O_{\text{F}2}-O_{\text{N}}-2O_{\text{F}1}-O_{\text{S}}=0$$

All experiments were performed in illuminated incubators (LRH-250-GS, Guangdong Medical Appliances Plant, Guangdong, China). The light intensity during photophase was approximately 1.97 W m^−2^ and the variation in temperature was ± 1°C.

### Inheritance of Diapause Duration (Intensity)

To understand whether a diapause-terminating condition has an influence on the inheritance of diapause intensity, diapause larvae (N, S, N × S, S × N strains) induced under LD 12:12 h at 22°C were divided into two groups 45 days after hatching. One group of the diapause larvae was transferred to a short photoperiod of LD 12:12 h (a diapause-inducing photoperiod) and a long photoperiod of LD 16:8 h (a diapause-averting photoperiod) at 25°C to test the effect of photoperiod on diapause maintenance and termination. The other group was placed at 5°C for 92 days in continuous darkness and then transferred to LD 16:8 h, 25°C to observe the diapause termination. Pupation was recorded every day until all of the diapausing individuals had pupated.

### Statistical Analyses

Statistical analyses were performed with SPSS 19.0 (SPSS Inc., IBM). All of the diapause incidence data were transformed by arcsin $\sqrt{x}$ prior to analysis. Logistic regression analysis was used to estimate the critical photoperiods for each group. One-way analysis of variance (ANOVA) and Tukey’s test were used to compare the differences in critical photoperiod among different groups. One-way ANOVA was used to determine whether there were significant differences in the incidence of diapause in different groups under each photoperiod at different temperatures. Pearson’s chi-squared test was used to test whether the results obtained under LD 11:13 h, LD 12:12 h, LD 13:11 h and LD 15:9 h for the F_2_, Bc-N, and Bc-S individuals fit the predictions of the additive model for these populations. Kruskal—Wallis test was used to determine whether the durations of diapause among different groups were significant.

## Results

### The Photoperiodic Response Curves for the Two Strains and Their F_1_ Progeny

**Fig 1:**
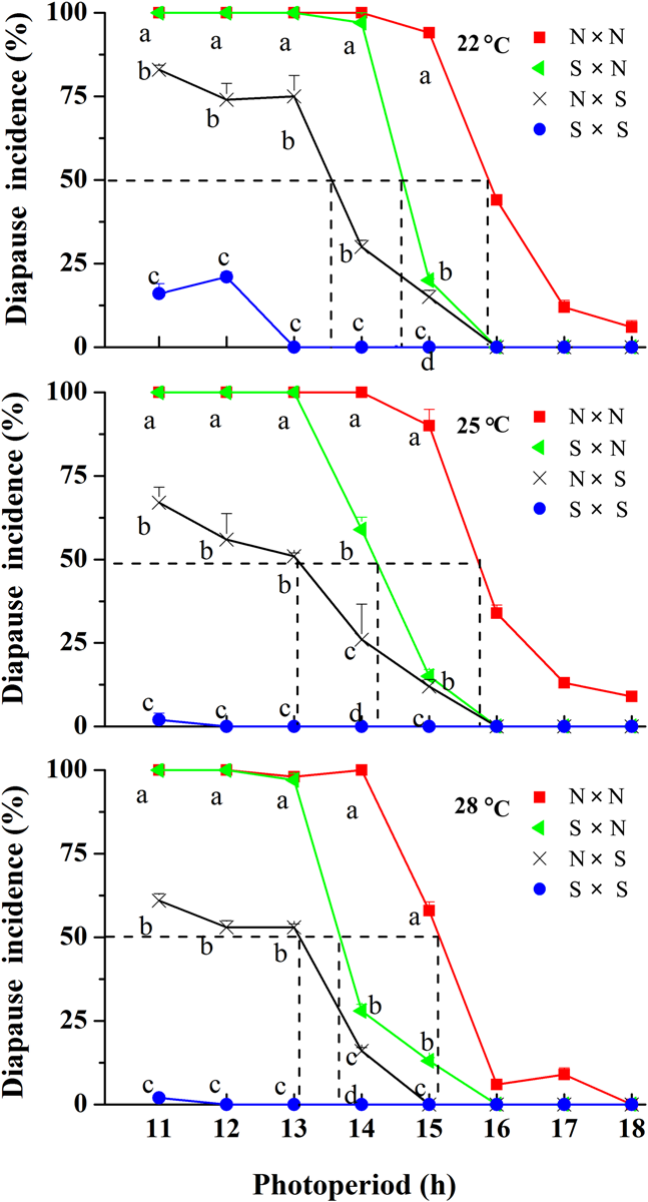
Photoperiodic response curves for the induction of larval diapause in *Ostrinia furnacalis* for the northernmost Harbin strain (N × N) (females shown on the left), the southernmost Ledong strain (S × S) and their F_1_ progeny (N × S, S × N) at 22, 25 and 28°C (n = 56–336). Error bars indicate the SD. Bars with different lowercase letters at the same photoperiod indicate significant differences in diapause incidence after one-way analysis of variance (*P* < 0. 05) (22°C, 11 h: *F*_3, 8_ = 3651.09, *P* ≤ 0.001; 12 h: *F*_3, 8_ = 1086.39, *P* ≤ 0.001; 13 h: *F*_3, 8_ = 1128.35, *P* ≤ 0.001; 14 h: *F*_3, 8_ = 395., *P* ≤ 0.001; 15 h: *F*_3, 8_ = 2451.82, *P*≤ 0.001; 25°C, 11 h: *F*_3, 8_ = 439.25, *P* ≤ 0.001; 12 h: *F*_3, 12_ = 645.80, *P* ≤ 0.001; 13 h: *F*_3, 8_ = 50612.06, *P* ≤ 0.001; 14 h: *F*_3, 8_ = 279.26, *P* ≤ 0.001; 15 h: *F*_3, 8_ = 1153.70, *P* ≤ 0.001; 28°C,11 h: *F*_3, 8_ = 515.59, *P* ≤ 0.001; 12 h: *F*_3, 8_ = 16782.44, *P* ≤ 0.001; 13 h: *F*_3, 8_ = 366.05, *P* ≤ 0.001); 14 h: *F*_3, 8_ = 4294.72, *P* ≤ 0.001; 15 h: *F*_3, 8_ = 1292.47, *P* ≤ 0.001).

Photoperiodic response curves for the induction of diapause from N and S strains and their F_1_ progeny (N × S, S × N) at 22, 25 and 28°C are shown in Fig. 1. The N strain showed a typical long-day response at all temperatures with a critical daylength (i.e., the daylength that elicits a 50% diapause response) of approximately15.88 h at 22°C, 15.72 h at 25°C and 15.14 h at 28°C (Table 1). Almost all S progeny developed without diapause at 25 and 28°C regardless of the photoperiod. However, the S strain showed a weak photoperiodic response at the lower temperature of 22°C. The incidence of diapause was 16.0% and 21.0% under the short daylengths of LD 11:13 h and LD 12:12 h, respectively. The F_1_ progeny (S × N and N × S) also showed a long-day response at all temperatures, but their critical daylengths were significantly shorter than those of the parental N strain (*P* < 0.05, Table 1). However, the incidence of diapause and the critical daylengths for the two F_1_ crosses were significantly different, with significantly higher diapause incidences and longer critical daylengths in the S × N crosses (with an N strain father) than those in the N × S crosses (with an S strain father) at all temperatures, indicating a sex linkage in the inheritance of diapause induction, with the male parent having more influence on the following F_1_ progeny. It is of interest to note that the incidence of diapause (97–100%) for the S × N crosses was the same as the N strain (98–100%) under the short daylengths of 11–13 h at all temperatures, indicating that diapause is completely dominant over non-diapause. However, the incidence of diapause for the F_1_ progeny was very low (15%–20% at 22°C; 12%–15% at 25°C; 0%–13% at 28°C) under the daylength of 15 h (close to the critical daylengths of the N strain) at all temperatures although the incidence of diapause for the N strain under the daylength of 15 h was still very high (94% at 22°C, 90% at 25°C and 58% at 28°C), showing that non-diapause is almost dominant under these conditions. At the daylengths of 16–18 h there was complete non-diapause at all temperatures in both crosses, even though some diapause is observed in the N strain. Thus the non-diapause trait is completely dominant at these long day-lengths. This implies that diapause induction is strongly influenced by interactions between F_1_ genotype and photoperiod.

**Table 1:** Critical daylength (h) of the northernmost Harbin (N) and the southernmost Ledong (S) strains of *Ostrinia furnacalis* and their F_1_ progeny based on their responses to various photoperiods at 22, 25 and 28°C.

Crosses (♀×♂)	Temperature (°C)		
	22	25	28
N	15.88 (15.81–15.95)^a^	15.72 (15.69–15.76)^a^	15.14 (15.04–15.24)^a^
S × N	14.53 (14.44–14.62)^b^	14.21 (14.14–14.27)^b^	13.68 (13.62–13.75)^b^
N × S	13.56 (13.50–13.13.63)^c^	13.09 (13.01–13.16)^c^	13.06 (13.03–13.10)^c^

Data are means and 95% confidence intervals; values followed by the same superscript lowercase letter within a column do not differ significantly at the 5% level by Tukey’s test after one-way analysis of variance.

### The Photoperiodic Response Curves for the Backcrosses and Reciprocal F_2_

**Fig 2:**
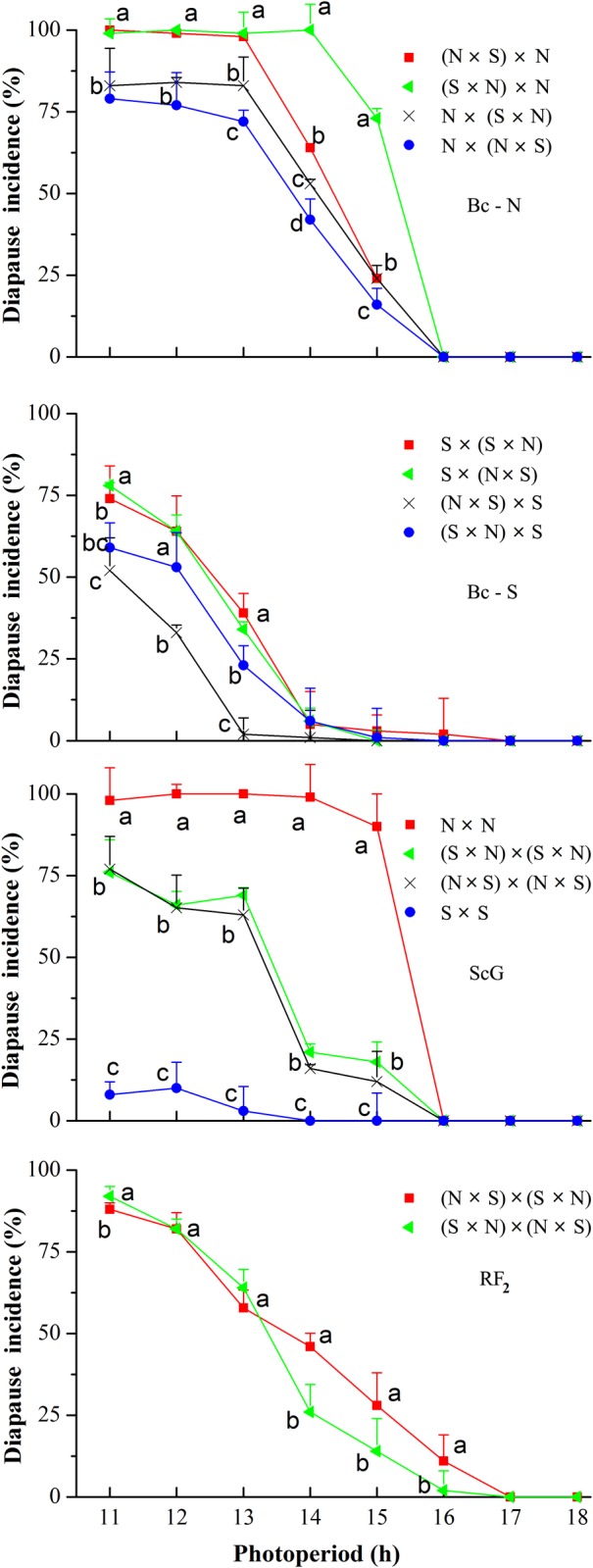
Photoperiodic response curves for the induction of larval diapause at 25°C in *Ostrinia furnacalis* for the backcross northernmost group (Bc-N), backcross southernmost group (Bc-S), the self-cross group (ScG) and the reciprocal F_2_ group (RF_2_) (n = 76–385). Error bars indicate the SD. Bars with different lowercase letters at the same photoperiod indicate significant differences in diapause incidence after one-way analysis of variance (*P* < 0. 05). Females show on the left for all the back-cross groups, self-cross group and reciprocal F_2_ group.

The photoperiodic response curves in the backcrosses and reciprocal F_2_ all showed a clear long-day response with different critical daylengths at 25°C (Fig. 2, Table 2). The critical daylengths were the longest in backcrosses to the N strain, followed by reciprocal F_2_ crosses and backcross to the S strain. There were significant differences in the critical daylength among different crosses (F _11, 24_ = 495.16, *P* < 0.05). The critical daylength was significantly different between the (S × N) × N cross (15.31 h) and the N × (N × S) cross (14.36 h) (*P* < 0.05, Fig. 2Bc-N and Table 2), between the (S × N) × S cross (12.25 h) and (N × S) × S cross (11.19 h) (*P* < 0.05, Fig. 2Bc-S and Table 2) and between the (N× S) × (S × N) cross (13.65 h) and (S × N) × (N × S) cross (13.36 h) (*P* < 0.05, Fig. 2RF_2_ and Table 2), showing a grandfather gene effect. The critical daylength was not significantly different between the (N × S) × (N × S) cross and the (S × N) × (S × N) cross (*P* > 0.05, Fig. 2ScG, Table 2).

**Table 2:** Critical day length of the reciprocal backcrosses of *Ostrinia furnacalis* based on their responses to various photoperiods at 25°C.

Crosses (♀×♂)	Critical photoperiod (h)
**Backcross to N**	
(N × N) × (N × S)	13.76 (13.57–13.95)^d^
(N × N) × (S × N)	14.11 (14.08–14.14)^C^
(N × S) × (N × N)	14.36 (14.32–14.39)^b^
(S × N) × (N × N)	15.31 (15.16–15.46)^a^
**Backcross to S**	
(S × S) × (N × S)	12.50 (12.43–12.57)^f^
(S × S) × (S × N)	12.39 (12.01–12.76)^fg^
(N × S) × (S × S)	11.19 (10.96–11.42)^h^
(S× N) × (S × S)	12.25 (12.04–12.45)^g^
**Reciprocal F**_**2**_	
(N × S) × (N × S)	13.28 (13.23–13.32)^e^
(S × N) × (S × N)	13.38 (13.36–13.41)^e^
(N× S) × (S × N)	13.65 (13.57–13.73)^d^
(S × N) × (N × S)	13.36 (13.23–13.48)^e^

Data are means and 95% confidence intervals, values followed by the same superscript lowercase letter do not differ significantly at the 5% level by Tukey’s test after one-way analysis of variance. N, the northernmost Harbin strain; S, the southernmost Ledong strain.

### Genetic Analysis of Diapause

**Fig 3:**
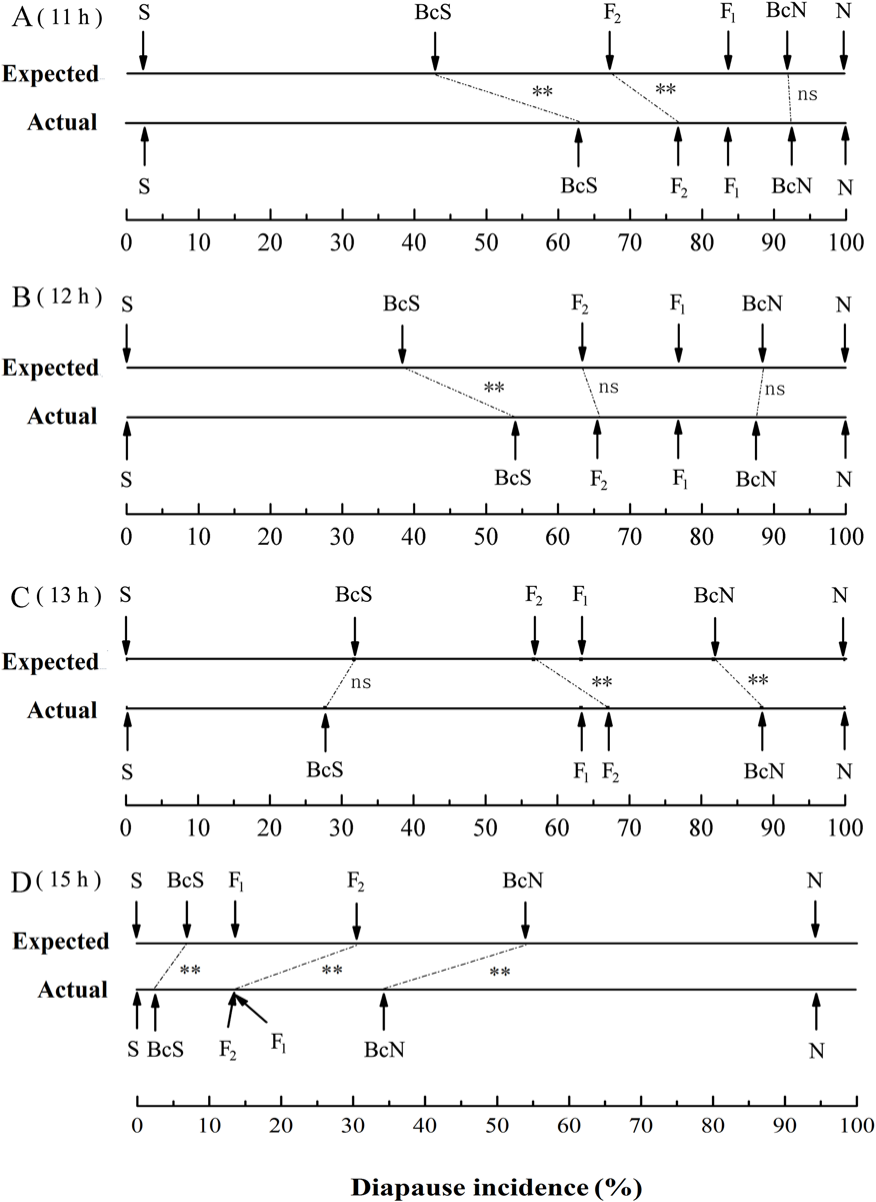
A comparison between the observed diapause incidence under LD 11:13 h (A), LD 12:12 h (B), LD 13:11 h (C) and LD 15:10 h (D) at 25°C among the progeny of crosses involving lines of *Ostrinia furnacalis* and the results predicted by an additive model with incomplete dominance (χ^2^-test, ns, not significantly different; **P < 0. 01) (also see Table 4–7). Bc-S, backcross to the southernmost Ledong strain; Bc-N, backcross to the northernmost Harbin strain.

The diapause incidence in progeny from all of the crosses under LD 11:13 h, LD 12:12 h, LD 13:11 h and LD 15:9 h at 25°C were used to evaluate the inheritance of diapause in this moth (Table 3, Fig. 3).

**Table 3:** Incidence of larval diapause of the parental strain, *F*_1_, *F*_2_ and backcross progeny in crosses and backcrosses of the N strain and S strain of *Ostrinia furnacalis* under different photoperiods at 25°C.

Cross (♀×♂)	LD 11:13 h		LD 12:12 h		LD 13:11 h		LD 15:9 h	
	Diapause% (N)	Expected value (additive model) (%)	Diapause % (N)	Expected value (additive model) (%)	Diapause % (N)	Expected value (additive model) (%)	Diapause % (N)	Expected value (additive model) (%)
Parental strains								
N × N	100 (171)		100 (381)		100 (102)		94.3 (336)	
S × S	2.4 (166)		0 (159)		0 (216)		0 (122)	
Reciprocal F1 hybrids								
N × S	67.1 (143)		56 (225)		51.4 (173)		11.8 (96)	
S × N	100 (140)		100 (201)		100 (56)		15.3 (111)	
F_1_ (Cum.)	83.4 (283)		76.5 (426)		63.3 (229)		13.7 (207)	
(N × S) × (N × S)	76.7 (313)		65.2 (385)		63.1 (111)		11.9 (261)	
(S × N) × (S × N)	76.1 (155)		65.6 (244)		68.8 (245)		17.9 (106)	
F_2_ (Cum.)	76.5 (468)	67.3**	65.4 (629)	63.3^ns^	67 (356)	56.7**	13.6 (367)	30.4**
Reciprocal backcrosses								
N × (N × S)	78.6 (145)		77 (239)		72.2 (169)		16.2 (191)	
N × (S × N)	82.9 (76)		84.1 (189)		82.7 (226)		24.1 (101)	
(N ×S) × N	100 (236)		99.2 (125)		98.1 (262)		24.4 (234)	
(S × N) × N	99.2 (125)		100 (135)		98.6 (145)		73 (174)	
BC-N (Cum.)	92.3 (582)	91.7^ns^	87.5 (688)	88.3^ns^	88.4 (802)	81.7**	34.2 (700)	54.0**
S × (N ×S)	77.9 (204)		66.1 (186)		33.7 (86)		0 (147)	
S × (S × N)	64.5 (242)		58.1 (296)		39.3 (298)		3.1 (223)	
(N × S) × S	52.4 (269)		33.1 (254)		2.2 (134)		2.9 (245)	
(S × N) × S	59.3 (246)		61.2 (242)		23.2 (125)		0.9 (115)	
BC- S (Cum.)	62.6 (961)	42.9**	53.9 (978)	38.3**	27.7 (643)	31.7^ns^	2.1 (730)	6.9**

**P < 0.01 (χ^2^), ns: not significantly different (χ^2^).

Bc-N, backcross to the northernmost Harbin strain; Bc-S, backcross to the southernmost Ledong strain.

**Table 4:** Influence of diapause-terminating conditions on the duration of diapause for each parent and their *F*_1_ progeny in *Ostrinia furnacalis*.

Cross (♀ × ♂)	Diapause-terminating photoperiod and temperature	Chilling period in 5°C (days)	N	Duration of diapause (d) ^a^
S × S	LD 12:12, 25°C	0	137	15.0 (3–82) A
N × S		0	141	16.2 (5–76) A
S × N		0	132	60.5 (14–106) B
N × N		0	67	67.6 (44–109) B
S × S	LD 16:8, 25°C	0	127	6.8 (1–30) A
N × S		0	123	8.9 (1–37) A
S × N		0	104	30.4 (11–77) B
N × N		0	97	41.0 (19–91) C
S × S	LD 16:8, 25°C	92	85	8.4 (4–41) A
N × S		92	178	17.5 (7–47) B
S × N		92	218	18.5 (8–41) B
N × N		92	94	37.9 (13–71) C

Diapause individuals were induced under LD 12:12 at 22°C, and then put under different conditions to terminate diapause 45 days after hatching. N, the northernmost Harbin strain; S, the southernmost Ledong strain.

^a^ Data are shown as the median (minimum-maximal value). Values followed by different letters are significantly different by Kruskal—Wallis test (*P* = 0.0001 < 0.01).

The simplest genetic model for testing these results assumes additive inheritance with no dominance. With no dominance the F_1_ progeny should show a diapause incidence intermediate to the two parental strains. In fact, the actual pooled F_1_ incidence of diapause (83.4% under LD 11:13 h; 76.5% under LD 12:12 h; 63.3% under LD 13:11 h) was significantly higher than the average diapause incidence of the parents (51.2% under LD 11:13 h; 50.0% under LD 12:12 h and LD 13:11 h) (11 h: χ ^2^ = 70.41, *P* < 0.01; 12 h: χ ^2^ = 70.90, *P* < 0.01; 13 h: χ ^2^ = 99.57, *P* < 0.01), suggesting that diapause is inherited additively with an incomplete dominance. By contrast, the actual pooled F_1_ incidence of diapause under LD 15: 9 h (13.7%) was significantly lower than the average diapause incidence of the parents (47.2%) (χ ^2^ = 69.43, *P* < 0.01), suggesting that non-diapause is partially dominant over diapause. These results further indicate that diapause is not merely promoted by the F_1_ genotype, but that diapause induction is also strongly influenced by interactions between the F_1_ genotype and photoperiod.

Results from the χ^2^ tests revealed that the patterns of inheritance for diapause capability were different under different photoperiods. The incidences of diapause under LD 12:12 h in F_2_ adequately fit the modified additive model (χ^2^ = 0.59, *P* = 0.444; Table 4 and Fig. 3B); whereas the incidence of diapause under LD 11:13 h, LD 13:11 h and LD 15:9 h in F_2_ were significantly different from the expectations (11 h: χ^2^ = 9.78, *P* < 0.01; 13 h: χ^2^ = 8.16, *P* < 0.01; 15 h: χ^2^ = 30.45, *P* < 0.01; Table 3, Fig. 3A, C, D), showing that the pattern of diapause was not additive. The data gathered on the diapause incidence in Bc-N under LD 11:13 h and LD 12:12 h fit an additive model (11 h: χ^2^ = 0.11, *P* = 0.75; 12 h: χ^2^ = 0.25, *P* = 0.62; Table 3, Fig. 3A, B). By contrast, the incidences of diapause under LD 13:11 h and LD 15:9 h in Bc-N did not fit an additive model (13 h: χ^2^ = 14.23, *P* < 0.01; 15 h: χ^2^ = 55.99, *P* < 0.01; Table 3, Fig. 3C, D). The data gathered on the diapause incidence in Bc-S under LD 13:11 h did fit an additive model (χ^2^ = 2.52, *P* = 0.113; Table 3, Fig. 3C); whereas the models under LD 11:13 h, LD 12:12 h and LD 15:9 h were not additive (11 h: χ^2^ = 75.36, *P* < 0.01; 12 h: χ^2^ = 47.53, *P* < 0.01; 15 h: χ^2^ = 19.72, *P* < 0.01; Table 3, Fig. 3A, B, D). These results clearly indicate that genetic and genetic-environmental interactions are involved in the induction of diapause.

### Inheritance of Diapause Intensity

**Fig 4:**
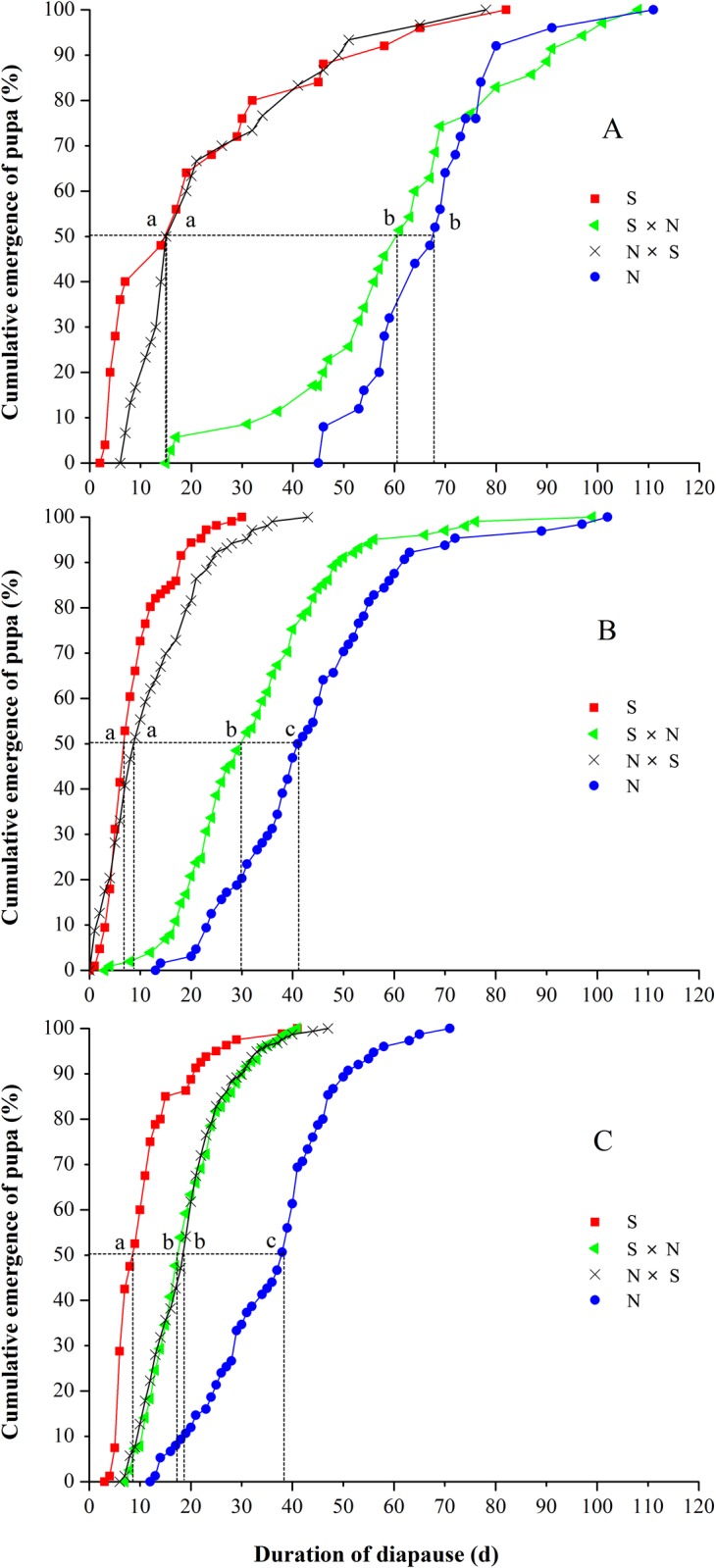
Termination of larval diapause in *Ostrinia furnacalis* for the northernmost Harbin strain (N × N) (females shown on the left), the southernmost Ledong strain (S × S), and their F_1_ progeny (N × S, S × N) (n = 67–218) at (A) LD 12:12 h (a diapause-inducing photoperiod), (B) LD 16:8 h (a diapause-averting photoperiod) at 25°C and (C) LD 16:8 h, 25°C after being placed at 5°C for 92 days in continuous darkness. Diapause was induced at 22°C, LD 12:12 h.

The cumulative percentage of pupation and the duration of diapause intensity under different diapause-terminating conditions (photoperiod, temperature, and chilling treatment) in N × N strains, S × S strains and their hybrids (N × S and S × N) are shown in Fig. 4 and Table 4, respectively. For each strain and cross, the three diapause-terminating conditions produced different patterns of inheritance. Under LD 12:12 h, the required number of days for pupation of 50% of the sample as well as the duration of diapause in the S × N cross was similar to the N strain, whereas the required number of days for 50% pupation and diapause duration in N × S cross was sample to the S strain, showing that the duration of diapause was determined by the male parents. There was a significant difference in the diapause duration among these treatments (Kruskal—Wallis test: χ^2^ = 61.05, *d*.*f*. = 3, *P* < 0.01; Fig. 4A, Table 4). Under LD 16:8 h, the required number of days for 50% pupation and the duration of diapause in the S × N cross was significantly shorter than in the N strain (*P* < 0.01), suggesting polygenic inheritance of diapause duration. By contrast, there were no significant differences between the N × S cross and the S strain (Kruskal—Wallis test: χ^2^ = 3.470, *d*.*f*. = 3, *P* = 0.062; Fig. 4B, Table 4). Under LD 16:8 h with 92 days of chilling, the required number of days for 50% pupation and the diapause duration of the N × S and S × N progeny was nearly the same, indicating that an equal genetic contribution to diapause intensity was made by each parent (Kruskal—Wallis test: χ^2^ = 0.012, *d*.*f*. = 3, *P* = 0.899; Fig. 4C, Table 4).

## Discussion

Results from photoperiodic response curves in *O*. *furnacalis* (Fig. 1) revealed that the induction of diapause was highly sensitive to photoperiod in the northernmost Harbin strain; nearly all larvae entered diapause under short daylengths of 11–13 h, even at 28°C. Such a high sensitivity to photoperiod also occurred in S × N crosses, in which the incidence of diapause (97%–100%) was nearly the same as the N strain (98%–100%) under the short daylengths of 11–13 h at all temperatures. However, in previous cross-mating experiments between the tropical S strain and the subtropical NC strain from Nanchang city (28°46′N, 115°50′E) under the short daylengths of 11 and 12 h at 25 and 28°C [19], the incidences of diapause in S × NC crosses (67.9%–88%) were significantly lower than those in the NC strain (89%–100%). The difference in the diapause capability in the two cross-mating experiments implies that the diapause gene is more powerful in the northernmost Harbin strain than it is in the southern NC strain of *O*. *furnacalis*. To the best of our knowledge, this is the first comparison of diapause capability between the northern and southern strains by cross-mating experiments. The high sensitivity to photoperiod in the N strain probably represents an adaptive evolution to the onset of diapause in field conditions. According to our field investigation in the Harbin region, nearly all first generation larvae of the N strain that hatched during July (the hottest month, with a mean temperature of 23.5°C, and daylength < 16 h) were destined to diapause regardless of the temperatures that prevailed in July [17]. Such a diapause-regulating mechanism may play an important role in the life history of the N strain because it ensures that larvae that hatch under high temperature conditions during the summer will enter diapause, thus avoiding the production of a subsequent generation that might fail in the region because of the ensuing low temperatures and the ripening and senescence of corn plants.

Whether the inheritance of diapause can be biased toward either parent depends on the species. The incidence of diapause has been found to be largely determined by the maternal line in the anise swallowtail, *Papilio zelicaon* [22], the blow fly, *Calliphora vicinia* [23,24] and the cabbage beetle, *Collaphellus bowringi* [25,26]; in which the reciprocal crosses of the diapause strain mothers produced a higher incidence of diapause among their offspring than did the non-diapause strain. Interestingly, most of the lepidopteran species typically indicate paternal effects rather than maternal effects, such as: the pink bollworm, *P*. *gossypiella* [7], the European corn borer, *Ostrinia*. *nubilalis* [27], the comma butterfly, *Polygonia c-album* [28], the Asian corn borer, *O*. *furnacalis* [19] and the cotton bollworm, *H*. *armigera* [11]; in which reciprocal crosses of high diapause strain fathers produced a higher incidence of diapause among their offspring than did low diapause strain fathers. The present cross-mating experiments in *O*. *furnacalis* further confirm the paternal effects in lepidopteran species. Especially, in that the S × N crosses yielded the same diapause rate as the N strain under the short daylengths of 11–13 h at all temperatures. Such paternal effect is intriguing, although the precise mechanism and relevance is unknown. Because Lepidoptera males are the homogametic sex (XX or ZZ) and females are the heterogametic sex (XY or ZW) [29], sex-linkage cannot readily explain such patterns [30]. In this context, it is of interest to note that an understanding of the genetic components of diapause may provide a genetic means to suppress pest populations [12,31]. As shown above, the N × S cross resulted in 85% of the individuals developing without diapause under the daylength of 15 h at 22°C (close to the critical daylength of 15.88 h for the N strain). According to field observations in the Harbin region, the adults from overwintering larvae emerged generally between late June and mid-July. The first generation of larvae hatched between early and late July and experienced the gradual shortening of natural daylengths, ranging from 16 h 38 min to 15 h 58 min (including twilight), and nearly all larvae entered diapause in response to the summer daylengths. If a large number of S strain males are released in the corn-fields in the Harbin region during late June and mid-July and allowed to mate with N females, most of the larval progeny that are produced by the cross would be forced to pupate and to emerge as adults during mid-August and early September. Consequently, larvae produced by these newly emerged adults would die with the onset of winter, and thus the number of adults available for mating and egg-laying would be reduced in the following early summer.

Our experimental results from F_1,_ F_2_ and backcross progeny revealed that the patterns of inheritance for diapause capability were obviously different under different photoperiods. Diapause phenotype in the S × N crosses was a completely dominant trait under the short daylengths of 11–13 h at 22, 25 and 28°C, evidenced by the 97%–100% diapause incidence; whereas non-diapause phenotype in the S × N crosses was a dominant trait under the daylength of 15 h at 22, 25 and 28°C in which more than 80% of individuals developed without diapause, although the N strain had a high incidence of diapause (94% at 22°C; 90% at 25°C; 58% at 28°C) (Fig. 1). The χ^2^ tests in *O*. *furnacalis* revealed that the photoperiod determines whether the inheritance of diapause fits an additive hypothesis. The incidences of diapause under LD 12:12 h in F_2_ crosses adequately fit the modified additive model (Table 3 and Fig. 3B); whereas the incidence of diapause under LD 11:13 h, LD 13:11 h and LD 15:9 h in F_2_ crosses did not fit an additive model (Table 3, Fig. 3A, C, D). Diapause incidence in Bc-N crosses under LD 11:13 h and LD 12:12 h fit an additive model (Table 3, Fig. 3A, B) but the incidence of diapause under LD 13:11 h and LD 15:9 h in Bc-N crosses did not fit an additive model (Table 3, Fig. 3C, D). The data of diapause incidence in Bc-S crosses under LD 13:11 h fit an additive model (Table 3, Fig. 3C); whereas the models under LD 11:13 h, LD 12:12 h and LD 15:9 h were not additive (Table 3, Fig. 3A, B, D). These results clearly indicate that a substantial proportion of the detectable variance for diapause capability depends on genetic-environmental interactions. Our results provide a better indication of the degree of polymorphism that exists in natural populations for diapause traits as well as suggesting that environmental conditions can strongly influenced the patterns of diapause inheritance. Our results also suggest that the patterns of dominance shift with varying daylengths in a manner which would help ensure that the “correct” developmental pathway is chosen even if there is some gene flow.

To date, few studies have reported the inheritance of diapause intensity, probably because of the difficulty and time required to collect the data. Reciprocal crosses that produced hybrids with a diapause intensity that was intermediate to parental values were found in the anise swallowtail, *Papilio zelicaon* [32], the fly *Drosophila triauraria* [33], the blow fly *C*. *vicinia* [34] and the cotton bollworm, *H*. *armigera* [35], suggesting that the duration of diapause was inherited in a quantitative manner. Furthermore, most of these crosses were performed under only one environmental condition. It is possible that the patterns of inheritance for diapause intensity may be different in another environment. The present study provides evidence that the patterns of inheritance for diapause intensity were strongly influenced by the diapause-terminating conditions in *O*. *furnacalis*. Under the short daylength of LD 12:12 h, the duration of diapause in reciprocal crosses (S × N and N × S) was determined by the male parent, i.e., diapause duration in S × N cross was similar to the N strain, whereas diapause duration in the N × S cross was similar to the S strain (Table 4). Under the long daylength of LD 16:8 h, diapause duration in the S × N cross was significantly shorter than in the N strain but no significant difference was found between the N × S cross and the S strain (Table 4). Under LD 16:8 h with 92 days of chilling, the duration of diapause in the N × S and S × N progeny was nearly the same (Table 4), showing that the duration of diapause was equally influenced by both parents. We believe this to be the first demonstration that the patterns of inheritance for diapause intensity are different under different diapause-terminating conditions.

All the results concerning diapause induction and termination in this study demonstrate that the inheritance of photoperiodic control of diapause in *O*. *furnacalis* is complicated and there is sufficient genetic variation upon which environmental factors can operate to influence the patterns of inheritance for diapause traits. Therefore, our results emphasize the importance of performing a cross-mating experiment under a range of environmental conditions.
